# Increased Sensitivity of Detection of *RASSF1A* and *GSTP1* DNA Fragments in Serum of Prostate Cancer Patients: Optimisation of Diagnostics Using OBBPA-ddPCR

**DOI:** 10.3390/cancers13174459

**Published:** 2021-09-04

**Authors:** Markus Friedemann, Friederike Horn, Katharina Gutewort, Lars Tautz, Carsten Jandeck, Nicole Bechmann, Olga Sukocheva, Manfred P. Wirth, Susanne Fuessel, Mario Menschikowski

**Affiliations:** 1 Institute of Clinical Chemistry and Laboratory Medicine, Carl Gustav Carus University Hospital, Technische Universität Dresden, Fetscherstr. 74, D-01307 Dresden, Germany; Markus.Friedemann@uniklinikum-dresden.de (M.F.); Lars.Tautz@mailbox.tu-dresden.de (L.T.); Friederike.Horn@tu-dresden.de (F.H.); Katharina.Gutewort@mailbox.tu-dresden.de (K.G.); Carsten.Jandeck@uniklinikum-dresden.de (C.J.); Nicole.Bechmann@uniklinikum-dresden.de (N.B.); 2 Department of Medicine III, University Hospital Carl Gustav Carus, Medical Faculty Carl Gustav Carus, Technische Universität Dresden, Fetscherstrasse 74, 01307 Dresden, Germany; 3 German Department of Human Nutrition Potsdam-Rehbruecke, Institute of Experimental Diabetology, 14558 Nuthetal, Germany; 4 German Center for Diabetes Research (DZD), 85764 München-Neuherberg, Germany; 5 School of Health Sciences, Flinders University of South Australia, Bedford Park, SA 5042, Australia; Olga.Sukocheva@flinders.edu.au; 6 Department of Urology, Carl Gustav Carus University Hospital, Technische Universität Dresden, Fetscherstr. 74, D-01307 Dresden, Germany; Manfred.Wirth@uniklinikum-dresden.de (M.P.W.); Susanne.Fuessel@uniklinikum-dresden.de (S.F.)

**Keywords:** liquid biopsy, cancer diagnostics, prostate cancer, circulating cell-free DNA (cfDNA), DNA methylation, cell-free tumour DNA, digital PCR

## Abstract

**Simple Summary:**

Prostate carcinoma (PCa) incidence rates have continued to increase over the last 3 decades worldwide and PCa is the most common cancer in men in Germany. Classical tumour markers exhibit unsatisfactory sensitivity and specificity, leading to unnecessary prostate biopsies and associated clinical complications. New, minimally invasive biomarkers with high sensitivity and specificity are required for an early diagnosis and a better prognosis. In the present study, we evaluated the performance of a previously developed analytical method to detect trace amounts of aberrant methylated tumour DNA. The optimised method which was developed in this study evaluates DNA methylation changes of *RASSF1A* and *GSTP1* in the blood of PCa patients at different stages compared to benign prostatic hyperplasia patients and healthy individuals. *RASSF1A* methylation analysis was found to be beneficial as a complementary biomarker for PCa diagnosis in combination with classical PCa markers. Both *RASSF1A* and *GSTP1* exhibited pathological DNA methylation levels in all metastatic PCa patients, which may improve early detection of PCa metastases and therapeutic outcomes.

**Abstract:**

Identification of aberrant DNA methylation is a promising tool in prostate cancer (PCa) diagnosis and treatment. In this study, we evaluated a two-step method named optimised bias-based preamplification followed by digital PCR (OBBPA-dPCR). The method was used to identify promoter hypermethylation of 2 tumour suppressor genes *RASSF1A* and *GSTP1* in the circulating cell-free DNA (cfDNA) from serum samples of PCa patients (*n* = 75), benign prostatic hyperplasia (BPH, *n* = 58), and healthy individuals (controls, *n* = 155). The PCa cohort was further subdivided into subgroups comprising (I) patients with Gleason Scores (GS) ≤ 7 (*n* = 55), (II) GS ≥ 8 (*n* = 10), and (III) patients with metastatic PCa diagnosis (*n* = 10). We found that *RASSF1A* methylation levels were significantly increased in all 3 PCa subgroups compared to the controls and BPH cohorts (*p* < 0.01 for all comparisons). Fractional abundances of methylated *GSTP1* DNA fragments were significantly increased in subgroup III of metastatic PCa patients (*p* < 0.001). *RASSF1A* methylation analysis was found to be beneficial as a complementary biomarker where further diagnostic validation is most crucial. In combination with free PSA, *RASSF1A* methylation status helps to identify PCa patients with GS ≥ 8 and grey-zone total PSA values between 2–10 ng/mL. In our study, PCR biases between 80–90% were sufficient to detect minute amounts of tumour DNA with high signal-to-noise ratios as well as high analytical sensitivity and specificity. Both *RASSF1A* and *GSTP1* exhibited strongly increased DNA methylation levels in all metastatic PCa patients. Our data indicates a superior sensitivity of epigenetic biomarker analyses in early detection of PCa metastases that should also help to improve PCa therapy.

## 1. Introduction

Reliable prognoses of malignant diseases strongly depend on the stage of the disease at diagnosis and whether metastasis is present. For an early diagnosis, and subsequently better chances of a positive prognosis, new biomarkers and analytical techniques with high sensitivity and specificity are required for examining minimal-invasive blood samples and non-invasive urine, sputum, or seminal plasma samples. These developments would enable more precise monitoring of disease progression as well as improved prognosis and screening. Current tumour markers are often detected at unsatisfactory levels of diagnostic sensitivity and specificity, indicating their suboptimal biomarker capacity for disease detection and stratification. An established parameter for early diagnosis of prostate carcinoma (PCa) is the prostate-specific antigen (PSA), which exhibits a high diagnostic sensitivity of 91%, but a low specificity of only 14% (2.5 ng/mL as cutoff) [1]. Hence, investigations carried out in recent years revealed a PSA-based over-diagnosis, which caused 65–70% unnecessary prostate biopsies [2,3,4,5]. Nearly 13% of screened men had false-positive results after 3 to 4 PSA-based screening rounds [6]. Additionally, 0.5–1.0% of prostate biopsies resulted in clinically important infections, bleeding, or urinary retention [6], underlining the limitations of the established diagnostic methods. On the other hand, 20% of patients with low PSA values between 2.6 and 4.0 ng/mL are diagnosed with PCa after prostate biopsy [7]. The Prostate Cancer Prevention Trial (PCPT) reported that 15% of men with normal serum PSA lower than 4 ng/mL and cancer-negative results after digital rectal examination had biopsy-detectable PCa [8]. Moreover, current PSA-based screening practices cannot reliably distinguish between indolent disease versus life-threatening cancer, which suggests that a certain number of men received unnecessary treatments [9,10]. Therefore, there is an urgent need for new analytical techniques and specific early biomarkers that allow better differentiation of PCa behaviours and improved prediction of clinical outcomes.

Epigenetic mechanisms such as DNA methylation play an important role in many physiological and pathophysiological processes including carcinogenesis [11,12,13,14,15]. Considering that changes in methylation pattern occur in early carcinogenesis, analyses of aberrant DNA methylation patterns have attracted substantial interest as a reliable biomarker for the initiation of cancer onset and monitoring of disease progression [11,12,13,14,15,16]. Consequently, aberrant DNA hypermethylation and associated silencing of tumour suppressor genes were suggested to be reliable biomarkers of cancer initiation and treatment outcomes [11,12,13,14,15]. Several different methods with varying analytical sensitivities were described and used for identification of changes in methylation patterns of selected DNA fragments in blood, urine and other human body fluids (as liquid biopsy) or smears, and tissue specimens [17,18,19,20,21,22].

Previously, we described a new technique named OBBPA-ddPCR consisting of an optimised bias-based preamplification (OBBPA) step followed by digital droplet PCR (ddPCR) [23]. The method was found to be suitable for more sensitive and specific identification of minute amounts of tumour DNA against a high background of wild-type DNA [23]. Using this method, 5 copies of methylated tumour DNA fragments were detected against a background of 700,000 copies of unmethylated wild-type DNA fragments. With a high signal-to-noise ratio, this method allows for an analytical sensitivity of 0.0007%.

The aim of the current study was to study the performance of 2 newly developed OBBPA-ddPCR-based assays for *GSTP1* and *RASSF1A* methylation analysis using blood serum samples from PCa patients (*n* = 75). GSTP1 and *RASSF1A* DNA promoter hypermethylation is detectable in different types of malignancies and its potential as diagnostic and prognostic biomarker is extensively described in literature [24,25,26,27,28,29]. The results were evaluated in comparison to the *GSTP1* and *RASSF1A* methylation status of healthy individuals (*n* = 155) as well as benign prostatic hyperplasia (BPH, *n* = 58) patients. PCa and BPH cohorts were selected to include patients with total PSA values between 2.0 and 10 ng/mL, where further diagnostic validation is most crucial.

## 2. Materials and Methods

### 2.1. Individual and Pooled Serum Samples

PCa patient serum samples (*n* = 90) with varying PSA levels were pooled to generate 2 sets of samples for method development and validation. Set 1 contained 5 pooled PCa samples (PCa 1–PCa 5), while set 2 contained 24 pooled PCa samples (PCa 1–PCa 24) with a serum volume of 5 mL (Tables S1 and S2). Similar procedure was performed with samples from healthy individuals (*n* = 73) to form 2 sets of serum samples (set 1 with 5 (Ctrl 1–Crtl 5) and set 2 with 15 samples (Ctrl 1–Crtl 15), 5 mL each). The grouping allows to isolate sufficient amounts of circulating cell-free DNA (cfDNA) for subsequent analysis (Tables S1 and S2). 

All individual serum samples (1–2 mL) from healthy individuals as well as PCa and BPH patients with PSA serum levels between 2.0 and 10.0 ng/mL were retrospectively and randomly selected and analysed (Tables S3–S5). Serum samples from BPH and PCa patients were extracted before prostate biopsy or radical prostatectomy after identification of tumour cells in a previous biopsy. Serum samples taken during a medical training for blood collection were used as healthy control samples. The samples were collected from students who signed a respective consent agreement. All PCa patients with metastases were already undergoing treatment such as hormonal, chemo- or radiotherapy at the time of blood sampling, except of 1 patient (the patient Meta 3 (Table S5) who had metastases at the time of PCa diagnosis. PCa was diagnosed using routine histopathological examination of the surgically removed tumour and/or biopsies. PCa and BPH patient and disease characteristics, including age, average total PSA and free PSA values, Gleason scores, and tumour stages are summarized in Table S4. The patients’ and healthy individuals sample analysis was approved by the Ethics Committee of the Technische Universität Dresden (Dresden, Germany).

### 2.2. Isolation of cfDNA from Blood Serum Samples

Isolation of cfDNA from 5 mL pooled serum samples or 1–2 mL individual serum samples was performed using the NucleoSnap DNA Plasma Kit (Macherey-Nagel, Düren, Germany) according to manufacturer’s instructions with an elution volume of 40 µL. All DNA samples were stored at −80 °C until bisulfite modification.

### 2.3. Bisulfite Modification of Isolated cfDNA

DNA concentration was determined using Quantus photometer and QuantiFluor dsDNA-System Kit (Promega, Mannheim, Germany) and bisulfite modification was conducted with EpiTect Fast DNA Bisulfite Kit (Qiagen GmbH, Düsseldorf, Germany) according to manufacturer’s instructions with an elution volume of 40 µL EB buffer. Samples were stored at −80 °C until analysis. 

### 2.4. Preparation of DNA Samples with a 50% Methylation Degree

Standard DNA samples were prepared with equal amounts of unmethylated and methylated control DNA copies (Qiagen GmbH) to quantify the PCR bias as previously described [23]. During the preamplification step, 150 copies of unmethylated and 150 copies of methylated DNA (given 50% methylation degree) were utilized, keeping the copy number below the copy per droplet (CPD) value of 6 in the following ddPCR procedure after 15 cycles of preamplification. 

### 2.5. Preamplification of Bisulfite-Modified DNA

Optimisation of preamplification condition was described elsewhere [23]. A temperature gradient of 50 °C to 63 °C was set up using the built-in function of the CFX thermal cycler (Bio-Rad Laboratories GmbH, München, Germany). MgCl_2_ was added to the master mix to reach final concentrations of 1.5, 2.5, 3.5, 4.5, 6.0, and 8.0 mM MgCl_2_ as indicated. The PCR reaction was conducted in a total volume of 25 µL including 0.625 U of HotStarTaqPlus (Qiagen) and 200 µmol/L of dNTPs. Primers were applied at a final concentration of 400 nmol/L. Primer sequences are listed in Table S6. The thermal cycling conditions were as follows: 95 °C for 5 min and 15 cycles at 94 °C for 10 s; different annealing temperatures as indicated for 30 s, followed by 72 °C for 30 s; and a final hold at 4 °C. All reactions were performed as duplicates with 2 µL of bisulfite modified patient cfDNA.

In each run, 10 ng bisulfite modified unmethylated (0%) and methylated (100%) standard DNA (Qiagen GmbH) were used as positive controls together with non-template. Genomic DNA without bisulfite modification were used as additional negative controls. 

### 2.6. DdPCR of Bisulfite-Modified DNA

All ddPCR analyses were performed using the QX100 Droplet Digital PCR System (Bio-Rad) according to the manufacturer’s instructions and as previously described [23]. Probe sequences were designed using OligoArchitect™ software from Sigma-Aldrich (Taufkirchen, Germany) as listed in Table S6. Probes were synthesized with BHQ-1 as fluorescence quencher at the 3′-end as well as 5′-FAM- for methylated sequences, or 5′-HEX-modifications for detection of unmethylated sequences. The optimal ddPCR annealing temperature for *RASSF1A* and *GSTP1* was 50.7 °C, which was determined in preliminary experiments using 0% and 100% methylated standard DNA controls (Qiagen). Primer and probes were used at final concentrations of 900 nmol/L and 250 nmol/L, respectively. The thermal cycling conditions included 95 °C for 10 min, 40 cycles of 94 °C for 30 s, and 50.7 °C for 1 min with a final 10 min hold at 98 °C. All methylation quantification experiments included no-template and genomic DNA controls. Data was analysed using the QuantaSoft version V1.6.6.0320 (Bio-Rad). To avoid over-representation of marginal amounts of methylated target DNA due to the stringent PCR bias and subsequent strong amplification of methylated DNA fragments in the preamplification step, we defined, that 2 separate positive OBBPA-ddPCR measurements per sample with more than 2 positive droplets are required to be considered as a pathological signal.

### 2.7. Data Analysis 

‘Center values’ were defined as means with standard deviations as error indication. Normal distribution was analysed using the Shapiro-Wilk test. Variance was estimated using F-test. Data was analysed using two-tailed and unpaired Student’s *t*-test (normally distributed, homoscedastic) or Mann-Whitney Rank Sum test (non-normally distributed) to calculate the indicated *p* values. Differences were considered as statistically significant at *p* < 0.05. Levels of significance were defined and indicated as *p* < 0.05 (*, #), *p* < 0.01 (**, ##), and *p* < 0.001 (***, ###).

The signal-to-noise ratio was defined in ddPCR-based experiments as the difference between FAM signal amplitudes (methylated DNA fragments) in PCa and control groups.

Receiver operating characteristic (ROC) curve analyses were conducted using the modules “roc_curve” and “auc” from the Scikit-learn library (Python 3.7.3).

The fractional abundance of methylated *RASSF1A* and *GSTP1* DNA fragments was defined as percentage of methylated DNA fragments and the sum of methylated and unmethylated DNA fragments of the respective biomarker.

## 3. Results

### 3.1. Analysis of Preamplification PCR Biases Using Primer Design, MgCl_2_ Concentration, and Annealing Temperature Optimisation

**Figure 1:**
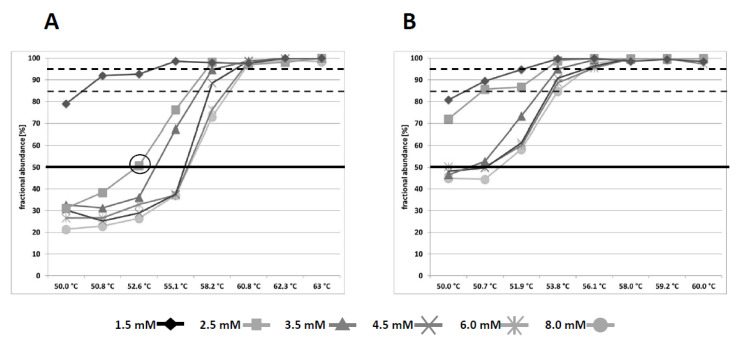
Analyses of *RASSF1A* and *GSTP1* PCR bias depending on pre-amplification conditions. Custom DNA standards were established comprising 150 copies of methylated and 150 copies of unmethylated DNA fragments for *GSTP1* and *RASSF1A*. The impact of varying magnesium chloride concentrations (1.5–8.0 mM) and annealing temperatures (50–63 °C) on the amplification efficiency of methylated and unmethylated *RASSF1A* (**A**) and *GSTP1* (**B**) template DNA was measured after 15 PCR pre-amplification cycles by subsequent ddPCR analysis. The percentages of methylated DNA fragments relative to the sum of all detected target sequences (fractional abundance) are illustrated.

To analyse PCR biases of *RASSF1A* (R-117bp) and *GSTP1* (G-116bp), custom DNA standards were established comprising 150 copies of methylated and 150 copies of unmethylated DNA fragments for *GSTP1* and *RASSF1A*. The impact of varying Mg^2+^ concentrations and annealing temperatures on the amplification efficiency of methylated and unmethylated template DNA was measured after 15 PCR preamplification cycles using subsequent ddPCR analysis. PCR bias was defined as percentage of methylated fragments relative to the total amount of target DNA fragments after 15 preamplification cycles (Figure 1). 

An annealing temperature above 60.8 °C or 58.0 °C resulted in an exclusive amplification of methylated DNA fragments for R-117bp and G-116bp, respectively (Figure 1A,B), independent of the corresponding Mg^2+^ concentration. A reduced annealing temperature led to a decreased amplification efficiency of methylated template, whilst unmethylated DNA fragment amplification was increased. Consequently, a PCR bias of ~80% and, thus, a residual amplification of unmethylated sequences was achieved through reduction of the annealing temperature to 50 °C for R-117bp and G-116bp under relatively low Mg^2+^ conditions of 1.5 mM. Furthermore, an increased Mg^2+^ concentration up to 8.0 mM caused a more efficient amplification of unmethylated DNA fragments and a subsequent reduction of the PCR bias in a temperature range between 50.0–58.2 °C and 50.0–53.8 °C for R-117bp and G-116bp, respectively (Figure 1). Hence, fine-tuning of Mg^2+^ concentration and annealing temperature enabled unbiased amplification of unmethylated and methylated target sequences for R-117bp (for instance, annealing temperature at 52.0 °C and corresponding Mg^2+^ concentration of 2.5 mM) and G-116bp (for instance, annealing temperature at 50.7 °C and corresponding Mg^2+^ concentration of 4.5 mM). Primers for R-117bp and G-116bp exhibited an intrinsic bias in favour of methylated DNA sequences due to inclusion of 2 or 3 5′-CpG sites, respectively. Notably, *RASSF1A* and *GSTP1* promoter regions are hypermethylated during prostate carcinogenesis [30,31] and, therefore, methylated DNA fragments are more relevant for sensitive PCa diagnostic methods. 

As previously described [23], a PCR bias between 80 and 90% was shown to be optimal with relatively high analytical sensitivity and specificity for the detection of minor amounts of methylated DNA fragments against excess amounts of unmethylated, wildtype DNA. An appropriate PCR bias and optimal overall PCR efficiency were achieved at an annealing temperature of 58.2 °C and 3.5 mM MgCl_2_ for R-117bp and 53.8 °C and 4.5 mM MgCl_2_ for G-116bp. Although, comparable PCR biases were detected by reducing the annealing temperature and Mg^2+^ concentrations, overall PCR amplification efficiency was reduced compared to the aforementioned conditions for R-117bp and G-116bp.

### 3.2. Analytical Sensitivity for Detection of Methylated RASSF1A and GSTP1 DNA Fragments in Pooled and Individual Serum Samples from PCa Patients and Healthy Controls

**Figure 2:**
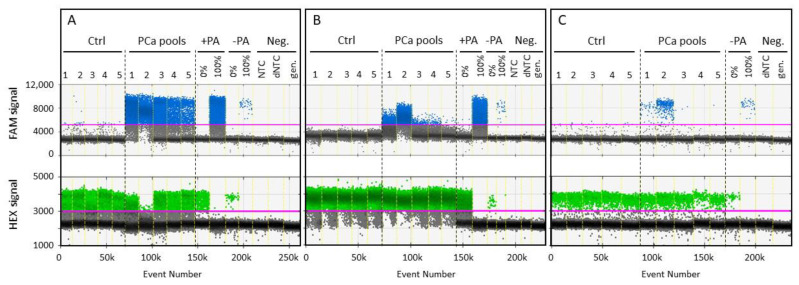
Identification of methylated and unmethylated *RASSF1A* DNA fragments in healthy controls and PCa serum pools. Thresholds for FAM signals specific for methylated (blue, upper row) and HEX signals for unmethylated (green, lower row) DNA sequences were 5200 and 3000 RFU, respectively. Serum pools of healthy individuals (Ctrl) and prostate carcinoma (PCa) patients were analysed by OBBPA-ddPCR (**A**), ddPCR after unbiased pre-amplification (**B**), and ddPCR alone (**C**). As control measurements, unmethylated (0%) and methylated (100%) DNA standards as well as no-template controls (dNTC without and NTC with pre-amplification) were analysed before (−PA) and after (+PA) pre-amplification. Genomic DNA standards without prior bisulfite treatment (gen.) were tested by ddPCR to ensure specific detection of bisulfite treated DNA.

The performance of R-117bp assay (3.5 mM MgCl_2_ and 58.2 °C) was evaluated using a first set as follows: 5 serum pools (each consisting of 5 samples) from healthy donors, 5 serum pools (each consisting of 5 samples) from PCa patients with increasing average PSA values (12.8 to 602.2 ng/mL) (summarized in Table S1). The maximum average fractional abundance of methylated *RASSF1A* fragments was 0.75% (Ctrl 1) in the tested 5 healthy serum pools. All 5 PCa pooled samples exhibited significantly higher amounts of methylated signals with high signal-to-noise ratios, which increased gradually with average PSA values of the different PCa serum pools (Figure 2A). Unbiased amplification with the same R-117bp primer pair was tested using adapted PCR conditions (2.5 mM MgCl_2_ and 52.0 °C) (Figure 2B). A 50% PCR bias was confirmed using DNA standards of methylated and unmethylated templates with comparable efficiencies. The lowest fractional abundance obtained after unbiased preamplification averaged 0.02% in PCa pool 5 (Table S1). However, in contrast to the OBBPA step, the signal-to-noise ratio, which signifies the difference between FAM signal amplitudes in PCa and control groups, was significantly lower when unbiased preamplification was used before ddPCR. Using the same pooled serums, positive duplicate measurements were detected in the 3 PCa pools with the highest average PSA values, except PCa pools 4 and 5 (Figure 2C). The data supports the superior sensitivity of the OBBPA-ddPCR technique compared to unbiased preamplification followed by ddPCR or ddPCR alone, as we have suggested previously [23]. 

**Figure 3:**
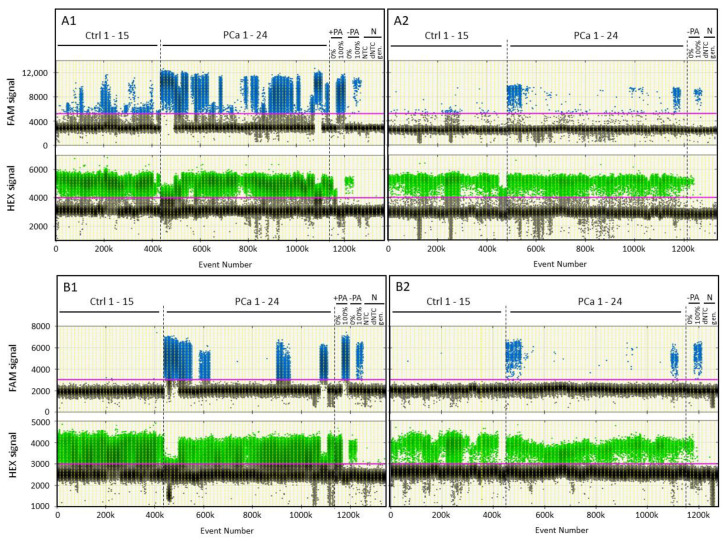
Diagnostic sensitivity of *RASSF1A* and *GSTP1* DNA methylation analyses under optimised PCR conditions. DNA methylation analyses were performed for *RASSF1A* (**A**) and *GSTP1* (**B**). Thresholds for FAM signals specific for methylated (blue, upper row) and HEX signals for unmethylated (green, lower row) DNA sequences were 5200/3000 RFU for *RASSF1A* and 3000/3000 RFU for *GSTP1*. Serum pools of healthy individuals (Ctrl) and prostate carcinoma (PCa) patients were analysed by OBBPA-ddPCR (**A1**,**B1**) and ddPCR alone (**A2**,**B2**). As control measurements, unmethylated (0%) and methylated (100%) DNA standards as well as no-template controls (dNTC without and NTC with pre-amplification) were analysed before (−PA) and after (+PA) pre-amplification. Genomic DNA standards without prior bisulfite treatment (gen.) were tested by ddPCR to ensure specific detection of bisulfite treated DNA.

Furthermore, we aimed to evaluate the diagnostic sensitivity of R-117bp assay under optimised PCR conditions. Additional 24 serum pools from PCa patients were analysed. The samples had a wide range of average PSA values between 1.3 to 406.4 ng/mL (summarized in Table S2) compared to serum pools of healthy donors (Figure 3). Low level of *RASSF1A* methylation was detected in healthy pools with a maximum average fractional abundance of 4.4%. Notably, 75% (18 out of 24) of analysed PCa serum pools and 73% (11 out of 15) of the pools with average PSA values below 10 ng/mL exhibited positive signals above the cutoff values of the healthy control group (Figure 3A1 and Table S2). In the same pooled controls or PCa samples, no G-116bp methylated target sequences were detectable in the control group, while 37.5% (9 out of 24) of analysed PCa pools showed positive signals with a strong signal-to-noise ratio (Figure 3B1 and Table S2). Only 4 specimens (26.7%) were positive among those 15 serum pools which had average PSA values below 10 ng/mL (Figure 3B1). 

The examination of the second set of specimens yielded the following data: *RASSF1A* ddPCR procedure alone (Figure 3A2) resulted in a lower overall diagnostic sensitivity of 25% (1.2% cutoff; 6 positive out of 24 patient pools) compared to 75% using OBBPA-ddPCR (18 positive out of 24 positive). The ddPCR procedure alone demonstrated lower diagnostic sensitivity for patients with PSA < 10 ng/mL (3 out of 15 patient pools) compared to OBBPA-ddPCR (11 out of 15). Diagnostic sensitivity of *GSTP1* ddPCR alone was also lower (0% cutoff; 7 positive out of 24 patient pools; Figure 3B2) compared to OBBPA-ddPCR for the entire patient cohort (9 out of 24). Lower sensitivity of *GSTP1* ddPCR was observed for serum pools from patients with PSA < 10 ng/mL (2 positive out of 15 patient pools) compared to OBBPA-ddPCR (4 positive out of 15).

**Figure 4:**
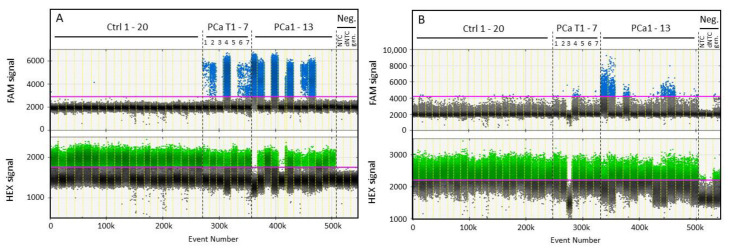
Methylation analyses of *GSTP1* DNA fragments in healthy controls and PCa serum pools. The DNA methylation status of *GSTP1* was analysed in serum samples of healthy individuals (Ctrl 1–20) and prostate carcinoma (PCa) patients at diagnosis (PCa 1–13) as well as in 1 PCa patient during PCa treatment (PCa T1–T7). The impact of PCR primer design on the analytical sensitivity and signal-to-noise ratio was demonstrated by comparing the OBBPA-ddPCR assay GSTP1-116bp (**A**) with an alternative GSTP1-120bp assay from literature (**B**). Thresholds for FAM signals specific for methylated (blue, upper row) and HEX signals for unmethylated (green, lower row) DNA sequences were 2900/1750 RFU for GSTP1-116bp and 4200/2200 RFU for GSTP1-120bp. As control measurements, unmethylated (0%) and methylated (100%) DNA standards as well as no-template controls (dNTC without and NTC with pre-amplification) were analysed before (−PA) and after (+PA) pre-amplification. Genomic DNA standards without prior bisulfite treatment (gen.) were tested by ddPCR to ensure specific detection of bisulfite treated DNA.

To reflect the importance of optimized primer design, the diagnostic sensitivity was measured using the G-116bp assay and compared to the GSTP1-120bp assay data (using primers as described previously [32]). The compared ratios are shown in Figure 4 for the set of 13 individual serum samples of PCa patients (the related information is summarized in Table S3) and 20 control serum samples from healthy individuals. The maximum fractional abundance of methylated G-116bp DNA fragments was 0.03% in the control samples, whereas methylated G-116bp positive signals were present in 53.8% (7 out of 13) of PCa serum samples (Figure 4A). To accent the increased sensitivity of detection, the results of 1 patient with advanced PCa (9 months follow-up; 7 blood sample collections) are also shown in Figure 4A. Although the unmethylated G-116bp background signal was relatively constant during the whole measurement period, highly variable levels of methylated *GSTP1* DNA fragments were detected in 5 out of 7 samples collected at the different time points during anti-cancer therapy. Compared to G-116bp, G-120bp signal quality, which signifies the difference between FAM signal amplitudes in PCa patients and healthy individuals at conditions of 2.5 mM Mg^2+^ and 50.7 °C, was decreased. Positive G-120bp signals were detected in 38.5% (5 out of 13) PCa patient samples and in 1 (time point #4) out of 7 samples during anti-cancer therapy.

### 3.3. Comparison of RASSF1A and GSTP1 DNA Methylation between Individual PCa, BPH, and Healthy Control Serum Samples

**Figure 5:**
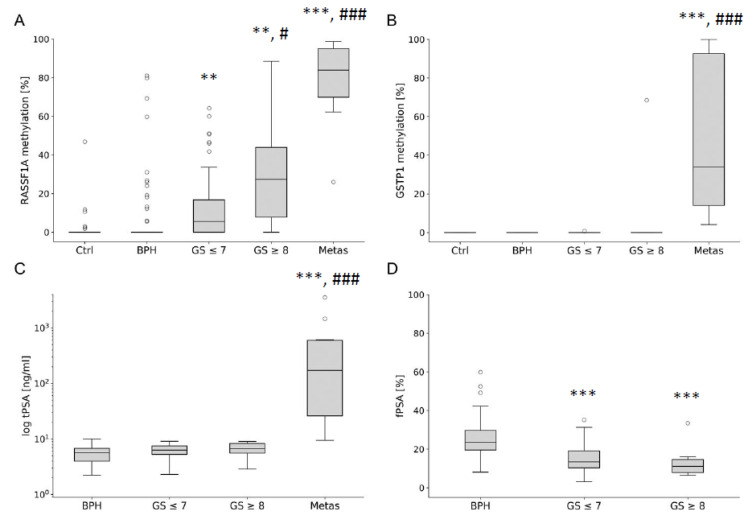
Methylation analyses of *RASSF1A* and *GSTP1* DNA fragments compared to tPSA and fPSA in healthy, BPH and different PCa subgroups. Box plots consist of the median as ‘center value’, the 25th and 75th percentiles as box edges, and the 10th and 90th percentiles as whisker boundaries. PCa and BPH cohorts were selected to include patients with tPSA values between 2.0 and 10 ng/mL, where further diagnostic validation is most crucial. Percentages of *RASSF1A* (**A**) and *GSTP1* (**B**) DNA methylation as well as tPSA (**C**) and fPSA (**D**) were analysed in BPH (*n* = 58), PCa patients with Gleason scores (GS) ≤ 7 (*n* = 55), GS ≥ 8 (*n* = 10), and metastatic prostate carcinoma (Metas, *n* = 10). (**A**,**B**) The healthy control group (Ctrl) was selected to be significantly younger than both the BPH patients and the PCa cohort to minimise the likelihood of undetected malignant disease. (**C**,**D**) Dashed lines represent cutoffs for tPSA (4 ng/mL) and fPSA (20%), which are used in routine diagnostics (Abbott). (**D**) No fPSA measurements were performed for metastatic PCa patient samples. The symbols * and # indicate significant differences compared to Ctrl and BPH (*) or low GS PCa group (#) with *p* < 0.05 (#), *p* < 0.01 (**), and *p* < 0.001 (***, ###).

To confirm the observed data, we analysed individual blood serum samples from healthy donors (*n* = 155), BPH (*n* = 58), and PCa patients (*n* = 75). The performance of optimised G-116bp and R-117bp assay was compared between samples with different total (tPSA), free PSA (fPSA), and PCa stages (summarized information is shown in Table S4 and Figure 5). The PCa cohort was subdivided into 3 subgroups (Figure 5) as follows: (I) patients with Gleason Scores (GS) ≤ 7 (*n* = 55), (II) GS ≥ 8 (*n* = 10), and (III) patients with metastatic PCa (*n* = 10). To minimise the likelihood of undetected malignant diseases, individuals significantly younger (24.0 ± 4.2, *p* < 0.001 for all comparisons) than both BPH and PCa cohorts were preselected for the healthy control group. PCa cohort I (65.0 ± 7.0 years) patients were significantly younger than BPH (69.5 ± 7.4 years, *p* < 0.01) and PCa cohort II (70.2 ± 7.5 years, *p* < 0.05) patients. PCa cohorts II and III patients showed no significant age differences compared to BPH. 

PCa and BPH cohorts included patients with tPSA values between 2.0 and 10.0 ng/mL, for whom further diagnostic validation is most crucial. No significant differences in tPSA levels were detected between BPH and PCa cohorts I and II, as well as between PCa cohort I and II (Table S5 and Figure 5). PCa cohort III exhibited increased tPSA levels (662.7 ± 1056.8, *p* < 0.001) compared to BPH and PCa cohorts I and II, as expected for metastatic PCa. Alternatively, fPSA was significantly decreased in PCa cohorts I (14.7 ± 6.9%) and II (12.9 ± 7.5%) compared to BPH patients (25.1 ± 10.0%, *p* < 0.001). No significant fPSA level differences between PCa cohorts I and II were observed (Table S5 and Figure 5).

The average fractional abundance of *RASSF1A* methylation was found to be significantly increased in PCa subgroups I (12.4%), II (29.8%), and III (78.1%) compared to the healthy controls (0.5%, *p* < 0.001) and BPH cohort (8.1%, *p* < 0.01). The highest fractional abundance of methylated *RASSF1A* DNA fragments was found in the metastatic PCa subgroup III, compared to PCa subgroup I (*p* < 0.001) and II (*p* < 0.01). No significant *GSTP1* methylation differences were detected between PCa subgroup I (0.013%) and II (6.9%) compared to controls or BPH cohort. Subgroup III of metastatic PCa patients exhibited a significantly higher fractional abundance of methylated *GSTP1* DNA fragments (48.4%), compared to healthy controls, BPH group, and PCa subgroups I and II (*p* < 0.001 for all comparisons). 

**Figure 6:**
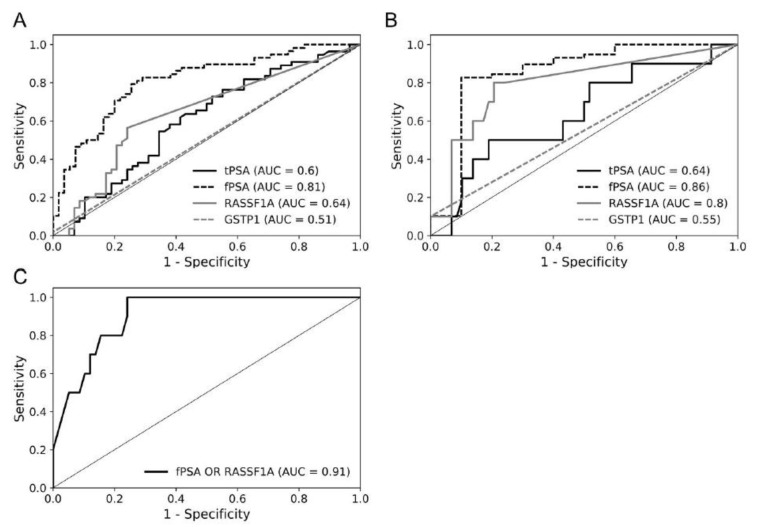
ROC curve analyses of *RASSF1A* and *GSTP1* DNA methylation as well as tPSA and fPSA in different PCa subgroups compared to BPH patients. PCa and BPH cohorts were selected to include patients with tPSA values between 2.0 and 10 ng/mL, where further diagnostic validation is most crucial. Area under the curve (AUC) values are indicated in figure legends. ROC curve analyses were performed by comparing PCa patient cohorts with low Gleason scores (GS) ≤ 7 ((**A**), *n* = 55) or high GS ≥ 8 ((**B**), *n* = 10) with the BPH patient group (*n* = 58), respectively. (**C**) Comparing high GS PCa patients with the BPH cohort, a combination of fPSA and *RASSF1A* methylation percentage resulted in an increased AUC of 0.91 compared to both markers alone.

Receiver operating characteristic (ROC) curve analyses were conducted. We compared diagnostic sensitivity (SEN), diagnostic specificity (SPE), and area under the curve (AUC) of the classical PCa markers tPSA and fPSA with the newly developed epigenetic biomarker assays G-116bp and R-117bp (Figure 6). PCa subgroup I was compared to BPH cohort. The test indicated that AUC values for fPSA (AUC = 0.81, 50% SEN and 89% SPE) and R-117bp (AUC = 0.64, 56% SEN and 76% SPE) were higher than those for tPSA (AUC = 0.60) and G-116bp (AUC = 0.51). AUC values were higher for fPSA (AUC = 0.86, 83% SEN and 90% SPE or 100% SEN and 40% SPE) and R-117bp (AUC = 0.80, 50% SEN and 93% SPE) when PCa subgroup II was compared with BPH. For the same comparison, AUC values were 0.64 and 0.55 for tPSA and G-116bp, respectively. Additionally, AUC value increased (AUC = 0.91, 50% SEN and 95% SPE or 100% SEN and 76% SPE) when fPSA and R-117bp were combined for comparison of PCa subgroup II and BPH group. 

## 4. Discussion

In the present study, 2 new OBBPA-ddPCR assays were developed to increase sensitivity of tumour-specific detection of methylated *RASSF1A* and *GSTP1* DNA fragments in serum samples of PCa patients. Our previous data confirmed the importance of a PCR bias between 80% and 90% in the preamplification step to generate a strong signal-to-noise ratio in the final ddPCR analysis. Primer pairs were selected for *RASSF1A* and *GSTP1* DNA fragments analyses to cover 2 and 3 5′-CpG sites, respectively. To achieve a PCR bias within the required range and reach a higher amplification efficiency, preamplification conditions with 3.5 mM MgCl_2_ at 58.2 °C or 4.5 mM MgCl_2_ at 53.8 °C were identified as particularly effective for the detection of methylated *RASSF1A* and *GSTP1* fragments, respectively. Assessment of *RASSF1A* methylation demonstrated that the real DNA methylation level could be directly determined when the PCR conditions support an unbiased preamplification. Such unbiased preamplification may be verified using unmethylated and completely methylated standard DNA samples. However, the disadvantages of unbiased preamplification included a reduced signal-to-noise ratio and lower analytical sensitivity compared to the optimised bias-based preamplification. Alternatively, the real DNA methylation degree can be calculated after OBBPA using DNA methylation standards with and without preamplification. Consequently, the efficiency of amplification of methylated and unmethylated DNA fragments during OBBPA can be determined and used for the re-calculation of the real DNA methylation degree in the analysed patient samples.

To reach diagnostic aims, it is important to detect smallest amounts of tumour DNA with high sensitivity and maximum analytical specificity of 100% over a strong background of unmethylated wildtype DNA fragments. Compared to ddPCR alone, methylated *RASSF1A* DNA fragments were detected in PCa serum pools 4 and 5 with the lowest PSA values using unbiased preamplification and OBBPA-ddPCR. This data indicates an increased sensitivity of the new method due to an additional amplification step prior to ddPCR. It is possible that the PCR reaction volume is incompletely encapsulated by lipid droplets during the ddPCR-based droplet-generating process. Preamplification may increase the probability of the transfer and detection of minute amounts of tumour-originating DNA fragments by ddPCR reaction, thus enhancing detection sensitivity. We found that used preamplification strategies resulted in the detection of all PCa serum pools. However, the signal-to-noise ratio, the indicator of discrimination between FAM positive signals in PCa patients and healthy individuals, was significantly increased during OBBPA-ddPCR approach. Our findings accent the importance of increased PCR bias (80–90%) for detection of small amounts of tumour DNA against a high physiological background [23]. 

Unbiased amplification of unmethylated and methylated DNA after bisulfite treatment may be required for unaltered DNA methylation level analyses, including whole genome bisulfite sequencing. However, it was noted that overrepresentation of either methylated or unmethylated DNA fragments during the initial amplification step may prevent accurate quantification of DNA methylation levels, representing a critical challenge for DNA methylation analysis [33,34]. Therefore, unbiased DNA amplification was achieved using adapted primer sequences, optimised Mg^2+^ concentration and annealing temperature as shown in the current study.

To collect proof-of-principle data, *RASSF1A* and *GSTP1* DNA methylation status was analysed in individual PCa serum samples with tPSA values between 2.0 and 10.0 ng/mL. This low tPSA level requires further diagnostic validation. To confirm diagnostic data, the methylated *RASSF1A* and *GSTP1* DNA levels in PCa serum samples were compared with those from healthy controls and BPH patients. Both fPSA and *RASSF1A* DNA methylation levels in PCa cohorts were significantly different from those in healthy individuals and BPH cohort (*p* < 0.01 for *RASSF1A* DNA methylation and *p* < 0.001 for fPSA). *RASSF1A* DNA methylation but not fPSA demonstrated a gradual and significant increase in the 3 analysed PCa cohorts (with GS ≤ 7, GS ≥ 8, and metastatic PCa). These findings suggest that *RASSF1A* DNA methylation analysis is a superior biomarker for tumour staging and prognosis, compared to tPSA and fPSA levels observed in the investigated cohort. Our data is supported by several recent studies [24,25,26,27]. Aberrant *RASSF1A* methylation levels in malignant tissues and cell-free circulating DNA samples were found to be a better diagnostic and prognostic biomarker in different types of malignancies, including hepatocellular [24] nasopharyngeal [25], and endometrial cancers [26]. Moreover, results of *RASSF1A* DNA methylation analysis of urine samples from PCa patients were discussed as potentially useful predictor of biochemical recurrence (combined with pathological staging data) in PCa patients with low Gleason score tumours [27]. Although the prognostic value of epigenetic biomarkers has been found to be highly efficient, there is a very limited number (we found only 1) of commercially available PCa tests. The available DNA methylation assay is based on tissue biopsy analysis of 3 genes (*APC*, *RASSF1*, *GSTP1*), and provides diagnostic rather than prognostic values [28,29]. 

In this study we found that *GSTP1* DNA methylation levels were increased in all metastatic PCa samples, while no significant *GSTP1* hypermethylation was detectable in the PCa cohorts I and II with GS ≤ 7 or GS ≥ 8, respectively. Accordingly, quantitative methylation-specific PCR-based analysis, reported by Brait et al. (2017) [35], demonstrated that 94% of tumour biopsies but 0% of matching serum samples of patients with early stage PCa showed *GSTP1* DNA hypermethylation. Additionally, TCGA Research Network (https://www.cancer.gov/tcga [36]) data analysis demonstrated significant *GSTP1* DNA hypermethylation levels in PCa tissues (GS 6–10) compared to corresponding normal prostate tissues (Figure S1). In this study, data may indicate on the limited stability of methylated *GSTP1* DNA fragments in blood stream, since unmethylated *GSTP1* DNA fragments were detected in all analysed patient samples used as internal controls. The observed effects may be influenced by high susceptibility of minute amounts of methylated *GSTP1* tumour DNA fragments to deoxyribonuclease (DNase) degradation in blood stream/serum. Accordingly, PCa patients exhibit lower blood DNase activity compared to healthy individuals, resulting in higher cfDNA concentrations [37]. DNase I treatment was associated with antimetastatic effects in animal tumour xenograft models, presumably via impairment of neutrophil extracellular traps (NET) formation [38,39,40,41]. DNase activity may be especially low in the blood of metastatic PCa patients, supporting the exclusive detection of methylated *GSTP1* DNA fragments in this PCa cohort. Alternatively, a limited release of methylated *GSTP1* DNA fragments from PCa tissues during the early phase of carcinogenesis may influence the detection levels, although the underlying mechanism requires further testing. Prognostic benefits of *GSTP1* methylation analysis were reviewed elsewhere [29,42].

ROC curve analysis revealed the benefits of fPSA measurements in addition to tPSA values in the diagnosis of investigated PCa cohorts in this study. Accordingly, results from Catalona et al. (1998) [43], Yilmaz et al. (2015) [44], and Caliskan (2017) [45] studies confirmed improved diagnostic sensitivity and specificity of fPSA/tPSA ratio for PCa patients with ambiguous tPSA values. Comparing PCa cohorts I and II with BPH patients, the highest AUC values were observed for fPSA, while a comparable AUC value was obtained for *RASSF1A* DNA methylation in PCa cohort II. Moreover, combined fPSA and *RASSF1A* DNA methylation data were associated with increased AUC values and better sensitivity/specificity in PCa cohort II. These findings confirm the benefits of DNA methylation analysis as complementary analysis method which may contribute and validate tPSA and fPSA measurements, improving PCa prognosis and therapy monitoring. 

Considering the limitations of the current study, we note that available material for *RASSF1A* and *GSTP1* methylation analyses was limited. The available 1–2 mL serum was divided into 4 parts for duplicate measurements with 2 biomarkers. Suggestively, an increased sample volume as well as multiplexed preamplification may further improve sensitivity of the investigated epigenetic biomarkers, especially in the detection of early-stage PCa. Multiplexed preamplification with inclusion of additional epigenetic biomarkers may improve diagnostic sensitivity/specificity of OBBPA-ddPCR without reduction of the available sample volume. Considering that the evaluation of maximum sensitivity and specificity of the epigenetic biomarkers for different PCa disease stages depends on sample material, volume and pre-analytics, larger investigations are warranted to confirm the data observed in this study. Furthermore, a prospective study involving the REMARK checklist [46,47] is needed to analyse the potential prognostic values of *RASSF1A* and *GSTP1* DNA methylation and the diagnostic performance of the described technique using larger number of patient blood samples.

## 5. Conclusions

2 new epigenetic marker assays for *RASSF1A* and *GSTP1* were established in this study. OBBPA-ddPCR technique was adapted and used to identify rare tumour DNA against a high background of wild-type DNA in serum from patients with prostate cancer. The collected data confirmed that PCR biases between 80–90% in combination with high preamplification efficiencies were sufficient for sensitive and specific detection of minute amounts of tumour DNA with high signal-to-noise ratios. Furthermore, *RASSF1A* methylation analysis was found to be beneficial as a complementary biomarker to fPSA values in the diagnosis of PCa patients with ambiguous tPSA values between 2–10 ng/mL and GS ≥ 8. For the group of patients with 2–10 ng/mL tPSA values, improved diagnostic validation is critical. Regarding metastatic PCa, both *RASSF1A* and *GSTP1* exhibited pathological DNA methylation levels in all PCa patients, supporting beneficial application of this epigenetic biomarker analysis for cancer therapy control and early detection of metastasis. 

## Data Availability

Data supporting reported results can be found online (no whomentary Tables S1–S5).

## Supplementary Materials

The following data is available online at https://www.mdpi.com/article/10.3390/cancers13174459/s1, Table S1, “Raw data for *RASSF1A* optimisation”; Table S2, “Analysis of the diagnostic sensitivity of *RASSF1A* and *GSTP1* methylation assays”; Table S3, “Raw data for *GSTP1* optimisation”; Table S4, “Characteristics of healthy blood donors (control), benign prostatic hyperplasia (BPH), and prostate carcinoma (PCa) patients included in the current study”; Table S5, “Patient information for benign prostate hyperplasia (BPH), prostate carcinoma (PCa), and metastatic PCa patients (Meta)”; Table S6, “Primer and probe sequences”; and Figure S1, “*RASSF1A* DNA methylation in prostate carcinoma biopsies compared to corresponding normal prostate tissue”.
