# Supplementary material for: Increased Sensitivity of Detection of RASSF1A and GSTP1 DNA Fragments in Serum of Prostate Cancer Patients: Optimisation of Diagnostics Using OBBPA-ddPCR
Source: Cancers (Basel). 2021 Sep 4;13(17):4459. doi: 10.3390/cancers13174459 (PMC8431466; doi:10.3390/cancers13174459)
Supplement: Supplementary file 1 [file cancers-13-04459-s001.zip › cancers-1310518-supplementary.pdf]

Supplementary Table S1. Raw data for RASSF1A optimisation (s. Fig. 2). Thresholds for FAM (specific for methylated DNA sequences) and HEX (specific for unmethylated DNA sequences) signals were 5200 and 3000 RFU, respectively. Serum pools of healthy individuals (Ctrl) and prostate carcinoma (PCa) patients were analysed by OBBPA-ddPCR, unbiased pre-amplification and ddPCR alone. Fractional abundance (FA) as well as methylated (M) and unmethylated (U) copies per 20µl ddPCR reaction are shown. SD – standard deviation, NTC – no template control, NA – not analysed.

sample	median PSA [ng/ml]	SD PSA [ng/ml]	OBBPA			unbiased pre-amplification			ddPCR alone		
			FA	copies/ddPCR		FA	copies/ddPCR		FA	copies/ddPCR	
				M	U		M	U		M	U
Ctrl 1			0.13	5	3860	0.00	0	69400	0.22	2	710
Ctrl 1			1.36	58	4240	0.00	0	60000	0.60	5	802
Ctrl 2			0.41	22	5460	0.00	0	86600	0.43	4	1038
Ctrl 2			0.32	24	7540	0.00	0	102200	0.30	3	898
Ctrl 3			0.00	0	3520	0.00	0	107200	0.35	4	1110
Ctrl 3			0.19	18	10020	0.00	0	92400	0.13	1	1030
Ctrl 4			0.28	13	4720	0.00	0	79000	0.13	1	940
Ctrl 4			0.33	18	5580	0.00	2	63800	0.14	1	982
Ctrl 5			0.09	3	3720	0.00	0	85000	0.60	5	902
Ctrl 5			0.38	18	4520	0.00	0	100600	0.16	1	864
PCa 1	602.2	198.2	93.02	62000	4660	3.06	3200	101400	9.10	70	700
PCa 1			94.46	45840	2680	3.11	3200	99600	10.20	80	706
PCa 2	244.1	49.0	99.94	87400	50	33.60	59600	118000	32.90	496	1008
PCa 2			98.31	106800	1840	37.20	60200	101600	27.70	432	1130
PCa 3	81.8	39.8	70.20	10980	4660	0.33	294	89200	0.50	4	678
PCa 3			80.50	10600	2560	0.45	388	85600	2.50	18	724
PCa 4	26.7	8.1	51.50	2200	2060	0.20	178	88600	0.00	0	214
PCa 4			56.10	2420	1900	0.15	136	88600	2.80	10	364
PCa 5	12.8	2.7	28.60	1576	3940	0.02	16	92800	0.40	2	442
PCa 5			18.20	872	3920	0.03	36	115200	0.00	0	520
0% (pre-amplified)			0.00	0	762	0.03	5	17200	NA	NA	NA

0% (pre-amplified)			0.39	3	776	0.03	5	17720	NA	NA	NA
100% (pre-amplified)			99.99	12540	1	100.00	16280	0	NA	NA	NA
100% (pre-amplified)			100.00	43040	0	100.00	17700	0	NA	NA	NA
0% (ddPCR)			0.00	0	76	0.00	0	72	0.00	0	76
0% (ddPCR)			0.00	0	78	4.00	3	70	0.00	0	78
100% (ddPCR)			100.00	72	0	100.00	90	0	100.00	72	0
100% (ddPCR)			100.00	76	0	100.00	62	0	100.00	76	0
NTC (pre-amplified)			-	0	0	-	2	2	NA	NA	NA
NTC (pre-amplified)			-	0	0	-	0	0	NA	NA	NA
NTC (ddPCR)			-	0	0	-	0	0	-	0	0
NTC (ddPCR)			-	0	5	-	0	0	-	0	5
genomic DNA			-	0	0	-	0	0	-	0	0
genomic DNA			-	0	0	0.00	0	186	-	0	0

Supplementary Table S2. Analysis of the diagnostic sensitivity of *RASSF1A* and *GSTP1* methylation assays (s. Fig. 3). Thresholds for FAM (specific for methylated DNA sequences) and HEX (specific for unmethylated DNA sequences) signals were 5200/3000 RFU for *RASSF1A* and 3000/3000 RFU for *GSTP1*. Serum samples of healthy individuals (Ctrl) and serum pools from prostate carcinoma (PCa) patients were analysed by OBBPA-ddPCR and ddPCR alone. Fractional abundance (FA) as well as methylated (M) and unmethylated (U) Copies per 20µl ddPCR reaction are shown. SD – standard deviation, NTC – no template control, NA – not analysed.

sample	median PSA [ng/ml]	SD PSA [ng/ml]	RASSF1A (OBBPA-ddPCR)			RASSF1A (ddPCR alone)			GSTP1 (OBBPA-ddPCR)			GSTP1 (ddPCR alone)		
			FA	copies/ddPCR		FA	copies/ddPCR		FA	copies/ddPCR		FA	copies/ddPCR	
				M	U		M	U		M	U		M	U
Ctrl 1			0.12	2	1340	0.50	1	258	0.00	0	1920	0.00	0	256
Ctrl 1			0.26	5	2040	1.60	4	260	0.00	0	1200	0.00	0	256
Ctrl 2			0.22	7	3180	0.70	1	188	0.00	0	2480	0.00	0	160
Ctrl 2			4.40	144	3100	0.00	0	188	0.00	0	2200	0.00	0	184
Ctrl 3			0.25	20	7540	0.50	4	764	0.00	0	12000	0.22	1	672
Ctrl 3			0.33	34	10220	0.00	0	692	0.00	0	5940	0.00	0	686
Ctrl 4			0.61	28	4400	0.70	4	532	0.00	0	2620	0.00	0	478
Ctrl 4			6.30	490	7320	0.30	2	504	0.00	0	6200	0.00	0	522
Ctrl 5			0.06	11	17540	0.24	3	1446	0.00	0	14600	0.00	0	1188
Ctrl 5			0.13	36	29180	0.32	4	1418	0.00	0	21380	0.00	0	1206
Ctrl 6			0.15	9	5820	0.26	1	486	0.00	0	4560	0.00	0	522
Ctrl 6			0.41	22	5220	0.29	1	494	0.00	0	5400	0.00	0	480
Ctrl 7			0.88	36	4080	0.00	0	330	0.00	0	13660	0.50	1	276
Ctrl 7			5.80	426	6940	1.80	6	304	0.02	2	8920	0.00	0	270
Ctrl 8			2.79	1680	58800	0.29	17	5840	0.00	0	55400	0.00	0	5000
Ctrl 8			0.88	604	68400	0.54	32	5920	0.01	2	32500	0.00	0	5160
Ctrl 9			0.14	86	61800	0.45	18	3900	0.00	0	37800	0.00	0	3300
Ctrl 9			0.10	82	81200	0.43	18	4100	0.00	0	55000	0.00	0	3400
Ctrl 10			0.02	2	9280	0.50	1	244	0.00	0	12200	0.00	0	176
Ctrl 10			0.13	8	6540	0.70	2	204	0.00	0	7600	0.00	0	194
Ctrl 11			6.00	90	1428	0.00	0	146	0.00	0	3060	0.00	0	136

Ctrl 11			2.40	46	1840	0.00	0	134	0.00	0	2000	0.00	0	114
Ctrl 12			8.40	120	1312	0.40	2	460	0.00	0	13420	0.00	0	412
Ctrl 12			0.48	42	8680	2.00	9	448	0.00	0	12720	0.00	0	280
Ctrl 13			0.52	112	21560	0.41	5	1120	0.00	0	21400	0.00	0	1020
Ctrl 13			0.48	114	23500	0.23	3	1108	0.00	0	40060	0.00	0	1032
Ctrl 14			1.65	208	12460	0.40	2	426	0.00	0	15020	0.00	0	360
Ctrl 14			0.93	92	9820	0.40	1	392	0.01	2	13420	0.00	0	382
Ctrl 15			0.00	0	318	0.00	0	168	0.20	2	798	-	0	5
Ctrl 15			41.50	254	358	0.00	0	154	0.00	0	932	-	0	3
PCa 1	406.4	248.4	99.99	20000000	1740	44.10	568	718	99.67	168000	566	31.30	320	702
PCa 1			98.76	192000	2420	42.60	604	816	99.42	38640	224	32.30	338	710
PCa 2	103.3	8.6	99.09	224000	2080	41.10	492	706	99.90	132800	140	33.00	352	714
PCa 2			99.99	20000000	1900	44.80	594	732	99.87	162000	218	34.50	336	638
PCa 3	75.9	3.4	78.30	27300	7580	2.10	16	720	62.30	9300	5620	1.60	10	636
PCa 3			74.00	22380	7880	3.70	26	700	57.70	9740	7160	2.00	13	620
PCa 4	32.3	16.7	74.50	11480	3920	1.20	6	512	54.80	1212	1000	0.40	2	378
PCa 4			81.70	10100	2260	1.10	6	506	67.80	2400	1146	0.90	3	362
PCa 5	21.4	0.1	0.00	0	966	0.00	0	428	0.00	0	1144	0.00	0	408
PCa 5			31.00	432	964	0.40	1	406	0.00	0	566	0.00	0	366
PCa 6	18.1	1.6	76.10	10980	3460	0.90	9	1048	28.10	314	802	0.00	0	780
PCa 6			48.90	2140	2220	0.80	9	1068	47.40	916	1016	0.00	0	770
PCa 7	14.3	1.2	26.30	1980	5560	0.00	0	846	23.50	742	2420	0.50	3	652
PCa 7			50.80	3980	3860	0.60	5	850	0.00	0	1412	0.25	2	644
PCa 8	11.5	1.2	0.35	50	14180	0.19	7	3900	0.00	0	3420	0.00	0	3020
PCa 8			5.17	748	13740	0.09	4	3800	0.00	0	4180	0.05	2	3100
PCa 9	10.2	0.3	0.26	15	5500	0.33	6	1760	0.00	0	838	0.00	0	1388
PCa 9			45.70	4580	5440	0.41	7	1692	0.00	0	1516	0.00	0	1406
PCa 10	8.9	0.6	0.38	11	2980	0.50	5	1022	0.00	0	1940	0.00	0	780
PCa 10			0.28	18	6540	0.45	5	1068	0.00	0	2300	0.00	0	744
PCa 11	7.8	0.3	0.76	66	8560	0.16	1	918	0.03	1	5700	0.00	0	752

PCa 11			0.13	8	6260	0.50	5	974	0.00	0	5920	0.00	0	770
PCa 12	7.2	0.1	2.30	5	206	0.70	1	174	0.00	0	530	1.10	2	142
PCa 12			13.00	32	218	0.00	0	178	0.00	0	268	0.00	0	148
PCa 13	6.5	0.6	26.30	1076	3020	0.50	3	534	0.00	0	1206	0.30	2	484
PCa 13			0.00	0	1314	0.50	3	504	0.00	0	1288	0.00	0	520
PCa 14	5.2	0.4	18.50	984	4340	0.49	5	1080	0.00	0	2540	0.16	2	1002
PCa 14			21.20	2000	7400	0.13	1	1168	0.00	0	4400	0.00	0	950
PCa 15	4.8	0.1	0.13	13	9520	1.10	5	426	0.00	0	12040	0.00	0	368
PCa 15			10.00	1196	10800	0.30	2	462	0.02	2	10880	0.00	0	340
PCa 16	4.4	0.2	0.08	10	13200	0.60	3	470	0.00	0	15420	0.00	0	284
PCa 16			12.90	1680	11340	0.30	2	472	0.00	0	13440	0.00	0	286
PCa 17	4.1	0.3	56.80	18800	14300	3.40	26	722	27.70	4640	12120	1.00	7	698
PCa 17			66.70	20120	10060	1.70	13	742	38.50	6480	10380	1.40	10	708
PCa 18	3.6	0.3	32.10	4640	9800	0.59	9	1558	6.66	982	13760	0.12	2	1360
PCa 18			50.00	12620	12640	1.20	18	1580	8.10	1284	14480	0.00	0	1386
PCa 19	3.2	0.1	28.80	2120	5240	1.10	3	280	0.02	2	9720	0.00	0	276
PCa 19			35.00	3540	6560	1.50	4	256	0.02	2	9440	0.00	0	248
PCa 20	2.7	0.2	0.23	26	11120	0.28	1	506	0.00	0	12380	0.00	0	356
PCa 20			14.30	1620	9680	0.50	3	520	0.00	0	13100	0.00	0	372
PCa 21	2.6	0.1	0.08	24	29620	0.60	7	1206	0.00	0	29420	0.00	0	1322
PCa 21			0.04	7	17760	0.44	6	1364	0.00	0	22180	0.12	1	1288
PCa 22	2.2	0.2	0.17	9	5680	0.70	3	494	0.00	0	6000	0.00	0	404
PCa 22			16.90	1900	9400	0.30	2	474	0.00	0	7900	0.00	0	338
PCa 23	1.3	0.3	99.98	20000000	3560	45.50	316	380	99.52	143000	686	40.00	228	340
PCa 23			99.98	20000000	5000	42.90	296	392	99.40	119200	714	35.70	218	392
PCa 24	1.4	0.6	0.51	17	3360	0.00	0	146	0.00	0	4340	0.00	0	120
PCa 24			40.70	2080	3020	0.00	0	114	0.00	0	1460	0.00	0	106
0% (pre-amplified)			0.00	0	2260	NA	NA	NA	0.00	0	10520	NA	NA	NA
0% (pre-amplified)			0.00	0	250	NA	NA	NA	0.00	0	15280	NA	NA	NA

100% (pre-amplified)			100.00	72400	0	NA	NA	NA	100.00	170000	0	NA	NA	NA
100% (pre-amplified)			100.00	83200	0	NA	NA	NA	100.00	108400	0	NA	NA	NA
0% (ddPCR)			2.40	3	112	0.00	0	64	0.00	0	490	0	0	198
0% (ddPCR)			1.00	1	130	0.00	0	42	0.00	0	490	0	0	204
100% (ddPCR)			100.00	140	0	100.00	62	0	100.00	514	0	96	232	9
100% (ddPCR)			100.00	88	0	97.00	52	1	100.00	432	0	100	226	0
NTC (pre-amplified)			-	0	0	NA	NA	NA	-	0	3	NA	NA	NA
NTC (pre-amplified)			-	0	0	NA	NA	NA	-	0	3	NA	NA	NA
NTC (ddPCR)			-	0	0	-	0	0	-	0	0	-	0	0
NTC (ddPCR)			-	0	1	-	0	0	-	0	0	-	0	1
genomic DNA			-	0	0	-	0	0	-	0	6	-	0	0
genomic DNA			-	0	0	-	0	0	-	0	2	-	0	0

Supplementary Table S3. Raw data for *GSTP1* optimisation. Thresholds for FAM (specific for methylated DNA sequences) and HEX (specific for unmethylated DNA sequences) signals were 2900/1750 RFU for GSTP1-116bp and 4200/2200 RFU for GSTP1-120bp. Serum samples of healthy individuals (Ctrl 1 - 20) and prostate carcinoma (PCa) patients at diagnosis (PCa 1 – 13) as well as one PCa patient during PCa treatment (PCa T1 – T7) were analysed by OBBPA-ddPCR and ddPCR alone. Fractional abundance (FA) as well as methylated (M) and unmethylated (U) Copies per 20µl ddPCR reaction are shown. NTC – no template control.

sample	PSA [ng/ml]	GSTP1-116bp			GSTP1-120bp		
		FA	copies/ddPCR		FA	copies/ddPCR	
			M	U		M	U
Ctrl 1		0.01	1	13880	0.00	0	29400
Ctrl 2		0.00	0	3800	0.05	7	15680
Ctrl 3		0.00	0	12560	0.00	0	21640
Ctrl 4		0.00	0	25740	0.00	0	35940
Ctrl 5		0.00	0	30200	0.00	0	48000
Ctrl 6		0.01	2	18640	0.00	0	39060
Ctrl 7		0.00	0	6360	0.02	4	21500
Ctrl 8		0.00	0	9800	0.01	2	41180
Ctrl 9		0.00	0	16700	0.00	0	33160
Ctrl 10		0.00	0	28740	0.00	0	36320
Ctrl 11		0.03	2	7640	0.00	0	38160
Ctrl 12		0.00	0	7400	0.01	2	32880
Ctrl 13		0.00	0	23700	0.00	0	41820
Ctrl 14		0.00	0	10080	0.02	4	19340
Ctrl 15		0.00	0	8060	0.02	6	27260
Ctrl 16		0.00	0	14900	0.00	0	46380
Ctrl 17		0.00	0	4860	0.02	4	22660
Ctrl 18		0.00	0	7660	0.00	0	12020
Ctrl 19		0.00	0	10940	0.00	0	14320
Ctrl 20		0.00	0	20200	0.00	0	29060
PCa T1	2071.1	5.10	166	3080	0.03	6	22160
PCa T2	1748.2	10.00	716	6400	0.02	2	12620

PCa T3	2311.2	0.06	2	3160	-	3	5
PCa T4	2978.9	91.31	26460	2520	0.32	54	16680
PCa T5	1739.0	0.00	0	8440	0.02	4	16740
PCa T6	1878.7	7.50	346	4280	0.00	0	11280
PCa T7	1871.0	6.10	512	7920	0.04	7	17240
PCa 1	1351.0	100.00	70000	2	37.50	2000	3340
PCa 2	344.2	83.30	5760	1152	3.72	620	16020
PCa 3	326.7	0.00	0	27060	0.00	0	34680
PCa 4	788.6	92.71	34140	2680	1.04	142	13520
PCa 5	151.1	0.00	0	28	0.03	3	12160
PCa 6	794.4	11.80	1020	7580	0.00	0	12960
PCa 7	107.1	59.80	9140	6120	0.01	2	21820
PCa 8	58.1	0.00	0	1480	0.22	16	7480
PCa 9	146.0	68.20	1366	636	2.02	196	9480
PCa 10	93.3	83.50	8300	1640	2.12	232	10720
PCa 11	87.8	0.00	0	4980	0.04	3	8280
PCa 12	84.2	0.00	0	5200	0.05	4	7080
PCa 13	76.4	0.00	0	2300	0.24	18	7720
NTC (pre-amplified)		-	0	0	0.00	0	70
NTC (ddPCR)		-	0	0	-	0	2
genomic DNA		-	0	0	0.70	7	1106

Supplementary Table S4. Characteristics of healthy blood donors (control), benign prostatic hyperplasia (BPH), and prostate carcinoma (PCa) patients included in the current study. Values are indicated as means with standard deviation. *NA - not available; no routine fPSA measurements were performed for metastatic PCa patient samples due to high tPSA values.

cohort	n	age [years]	mean tPSA [ng/ml]	mean fPSA [%]	Gleason score	tumour stage
Controls	155	24.0 ± 4.2	-	-	-	-
BPH	58	69.5 ± 7.3	5.6 ± 2.0	25.1 ± 10.0	-	-
PCa	65	65.8 ± 7.3	6.3 ± 1.8	14.5 ± 7.0	GS 6 (n=8), GS 7 (n=47), GS 8 (n=7), GS 9 (n=3)	cT1 (n=18), cT2 (n=1), pT2 (n=30), cT3 (n=1), pT3 (n=15)
Metastatic PCa	10	71.9 ± 11.2	662.7 ± 1056.8	NA*	GS 8 (n=1), GS 9 (n=7), GS 10 (n=2)	cT3 (n=4), pT3 (n=3), cT4 (n=2), pT4 (n=1)

Supplementary Table S5. Patient information for benigne prostate hyperplasia (BPH), prostate carcinoma (PCa), and metastatic PCa patients (Meta).

patient	age [years]	total PSA [ng/ml]	free PSA [%]	tumour stage	Gleason score	*RASSF1A* methylation [%]	*GSTP1* methylation [%]
BPH 1	70	9.97	10.73	-	-	0	0
BPH 2	62	9.66	23.29	-	-	0	0
BPH 3	69	9.49	13.38	-	-	81	0
BPH 4	67	9.27	42.29	-	-	0	0
BPH 5	73	8.72	27.29	-	-	5.9	0
BPH 6	80	8.58	20.4	-	-	0	0
BPH 7	70	8.21	32.76	-	-	0	0
BPH 8	76	8.16	22.92	-	-	0	0
BPH 9	66	8.08	12	-	-	0	0
BPH 10	56	7.87	20.08	-	-	0	0
BPH 11	69	7.7	23.77	-	-	0	0
BPH 12	67	7.37	18.05	-	-	0	0
BPH 13	67	7.29	19.34	-	-	0	0
BPH 14	65	7.03	22.62	-	-	0	0
BPH 15	63	6.82	15.54	-	-	0	0
BPH 16	61	6.70	21.79	-	-	0	0
BPH 17	68	6.65	22.71	-	-	0	0
BPH 18	71	6.60	28.48	-	-	59.8	0
BPH 19	75	6.46	22.76	-	-	0	0
BPH 20	67	6.42	9.66	-	-	0	0
BPH 21	63	6.14	26.06	-	-	0	0
BPH 22	71	6.08	49.18	-	-	0	0
BPH 23	57	5.99	29.88	-	-	0	0
BPH 24	67	5.91	12.01	-	-	0	0
BPH 25	75	5.80	33.19	-	-	0	0
BPH 26	76	5.78	19.72	-	-	31.1	0

BPH 27	66	5.77	31.89	-	-	0	0
BPH 28	64	5.74	26.83	-	-	0	0
BPH 29	73	5.73	35.43	-	-	0	0
BPH 30	71	5.54	28.16	-	-	0	0
BPH 31	74	5.38	20.07	-	-	0	0
BPH 32	73	5.29	36.67	-	-	12.1	0
BPH 33	77	5.20	26.73	-	-	0	0
BPH 34	83	5.05	29.31	-	-	25.9	0
BPH 35	69	5.00	28.2	-	-	26.8	0
BPH 36	67	4.93	21.7	-	-	19.2	0
BPH 37	74	4.61	26.9	-	-	0	0
BPH 38	61	4.55	18.46	-	-	0	0
BPH 39	69	4.43	15.12	-	-	0	0
BPH 40	61	4.34	20.05	-	-	0	0
BPH 41	82	4.28	32.48	-	-	0	0
BPH 42	66	4.08	19.32	-	-	0	0
BPH 43	84	4.04	20.79	-	-	0	0
BPH 44	82	3.95	8.1	-	-	5.5	0
BPH 45	59	3.92	30.1	-	-	13.2	0
BPH 46	73	3.89	59.9	-	-	0	0
BPH 47	77	3.77	25.2	-	-	0	0
BPH 48	58	3.70	9.19	-	-	0	0
BPH 49	82	3.67	14.71	-	-	0	0
BPH 50	85	3.65	24.11	-	-	18.1	0
BPH 51	68	3.17	18.93	-	-	24	0
BPH 52	66	3.08	19.81	-	-	0	0
BPH 53	62	3.06	33.01	-	-	0	0
BPH 54	51	2.75	25.82	-	-	0	0
BPH 55	71	2.66	33.08	-	-	0	0
BPH 56	69	2.64	28.79	-	-	69.2	0

BPH 57	66	2.25	52.44	-	-	79.8	0
BPH 58	76	2.21	32.13	-	-	0	0
PCa 1	76	9.1	6.9	T1	GS 3+4=7	5.1	0
PCa 2	67	9.08	8.92	T3	GS 3+4=7	0	0
PCa 3	72	9.04	7.85	T2	GS 4+5=9	34.2	0
PCa 4	72	8.97	12.04	T3	GS 3+4=7	41.8	0
PCa 5	64	8.95	15.64	T2	GS 3+4=7	5.4	0
PCa 6	53	8.63	10.31	T1	GS 3+4=7	0	0
PCa 7	77	8.58	8.04	T3	GS 3+5=8	13.2	68.5
PCa 8	62	8.56	6.43	T2	GS 3+4=7	18	0
PCa 9	56	8.49	6.48	T2	GS 3+4=7	0	0
PCa 10	61	8.43	10.44	T3	GS 4+3=7	9.2	0
PCa 11	72	8.39	6.08	T3	GS 3+4=7	0	0
PCa 12	84	8.32	11.54	T3	GS 4+4=8	6	0
PCa 13	60	8.28	27.5	T1	GS 3+4=7	0	0
PCa 14	68	8.26	7.87	T3	GS 3+4=7	17	0
PCa 15	65	8.15	6.38	T2	GS 3+5=8	0	0
PCa 16	67	6.31	4.7	T1	GS 3+3=6	64.2	0
PCa 17	52	7.85	8.8	T3	GS 3+4=7	0	0
PCa 18	65	7.67	13.3	T3	GS 4+3=7	51.1	0
PCa 19	64	7.63	15.33	T1	GS 4+4=8	88.5	0
PCa 20	80	7.56	12.17	T2	GS 4+3=7	20.7	0
PCa 21	50	7.52	9.6	T3	GS 3+4=7	0	0
PCa 22	67	7.14	15.41	T2	GS 3+4=7	0	0
PCa 23	67	7.00	14.29	T2	GS 3+4=7	0	0
PCa 24	68	6.96	19.8	T2	GS 3+4=7	60	0
PCa 25	55	6.95	16.4	T2	GS 3+3=6	0	0
PCa 26	65	6.81	23.49	T1	GS 3+3=6	0	0
PCa 27	72	6.68	35.18	T1	GS 3+4=7	11.1	0
PCa 28	71	6.57	12.5	T1	GS 3+4=7	0	0

PCa 29	69	6.5	31.23	T3	GS 3+4=7	0	0
PCa 30	57	6.38	6.74	T2	GS 3+4=7	16.4	0
PCa 31	65	6.29	18.28	T2	GS 3+3=6	0	0
PCa 32	60	6.29	4.29	T2	GS 3+4=7	27.5	0
PCa 33	56	6.26	12.3	T1	GS 3+4=7	0	0
PCa 34	65	6.23	3.05	T1	GS3+4=7	46.6	0
PCa 35	61	6.17	13.3	T1	GS 4+3=7	0	0
PCa 36	57	6.08	11.8	T2	GS 3+4=7	0	0
PCa 37	72	6.07	20.59	T2	GS 3+4=7	7	0
PCa 38	67	5.85	16.07	T2	GS 3+4=7	6.1	0
PCa 39	75	5.81	18.59	T3	GS 4+3=7	0	0
PCa 40	83	5.80	7.59	T2	GS 3+4=7	25.9	0
PCa 41	79	5.79	16.06	T1	GS 4+5=9	20.4	0
PCa 42	70	5.76	10.94	T3	GS 4+3=7	0	0
PCa 43	67	5.69	19.51	T1	GS 3+3=6	5.5	0
PCa 44	64	5.65	13.8	T1	GS 3+3=6	27.6	0
PCa 45	62	5.62	7.83	T2	GS 4+5=9	44.4	0
PCa 46	60	5.52	12.3	T2	GS 4+4=8	0	0
PCa 47	69	5.43	10.31	T2	GS 3+4=7	9.4	0
PCa 48	59	5.39	11.32	T3	GS 3+4=7	13.6	0
PCa 49	66	5.27	26.94	T1	GS 3+3=6	13.5	0
PCa 50	63	5.22	12.07	T2	GS 3+4=7	6.2	0
PCa 51	71	4.74	22.78	T2	GS 3+4=7	5.7	0.73
PCa 52	65	4.63	21.6	T2	GS 3+4=7	3.4	0
PCa 53	60	4.63	12.74	T2	GS 3+4=7	45.97	0
PCa 54	68	4.47	10.51	T3	GS 4+4=8	42.1	0
PCa 55	65	4.32	13.43	T2	GS 3+4=7	0	0
PCa 56	60	4.23	23.4	T2	GS 4+3=7	0	0
PCa 57	58	4.19	13.84	T2	GS 3+4=7	0	0
PCa 58	53	4.02	14.93	T2	GS 3+4=7	13.4	0

PCa 59	72	3.90	20	T2	GS 4+3=7	7.2	0
PCa 60	65	3.3	16.36	T1	GS 4+3=7	33.6	0
PCa 61	75	3.26	25.15	T1	GS 3+4=7	12.9	0
PCa 62	59	3.08	16.56	T1	GS 3+3=6	0	0
PCa 63	71	2.87	33.45	T3	GS 4+4=8	49.6	0
PCa 64	65	2.48	12.9	T2	GS 3+4=7	0	0
PCa 65	74	2.29	23.89	T2	GS 3+4=7	50.7	0
Meta 1	70	9.45	11.4	T4	GS 5+5=10	69.5	46.9
Meta 2	83	3558	-	T3	GS 4+5=9	95.1	75.2
Meta 3	81	1468.8	-	T3	GS 4+4=8	95.7	99.9
Meta 4	67	612.4	-	T3	GS 4+5=9	95.1	100
Meta 5	71	544.8	-	-	-	79.6	20.8
Meta 6	58	237	-	T3	GS 5+5 = 10	88.2	98.4
Meta 7	52	107.1	-	T1	GS 4+5=9	98.7	6.4
Meta 8	91	66.00	-	T3	GS 5+4=9	26	4.1
Meta 9	79	12.6	-	T3	GS 5+4=9	62.2	11.7
Meta 10	67	11.2	-	T4	GS 4+5=9	71	20.6

Supplementary Table S6. Primer and probe sequences. Primers for the GSTP1-120bp assay were published by Tserga *et al.* (2012).

assay	forward primer (5' → 3')	probe (5' → 3')<br>(methylated / unmethylated)	reverse primer (5' → 3')	amplicon size [bp]
RASSF1A-117bp	GTTTGTTAGCGTTTAAAGTTAG	[FAM]-CGCCCAACGAATACCAACTCCCG-[BHQ1]<br>[HEX]-CACCCAACAAATACCAACTCCCA-[BHQ1]	AATACGACCCTTCCCAAC	117
GSTP1-116bp	ATCGTAGCGGTTTTAGGGAAT	[FAM]-CGCAACGAAATATACGCAAC-[BHQ1]<br>[HEX]-CACAACAAAATATACACAAC-[BHQ1]	TCCCCAACGAAACCTAAAAA	116
GSTP1-120bp	GTGAAGCGGGTGTGTAAGTTT	[FAM]-CGCAACGAAATATACGCAAC-[BHQ1]<br>[HEX]-CACAACAAAATATACACAAC-[BHQ1]	TAAACAAACAACAAAAAAAAAACC	120

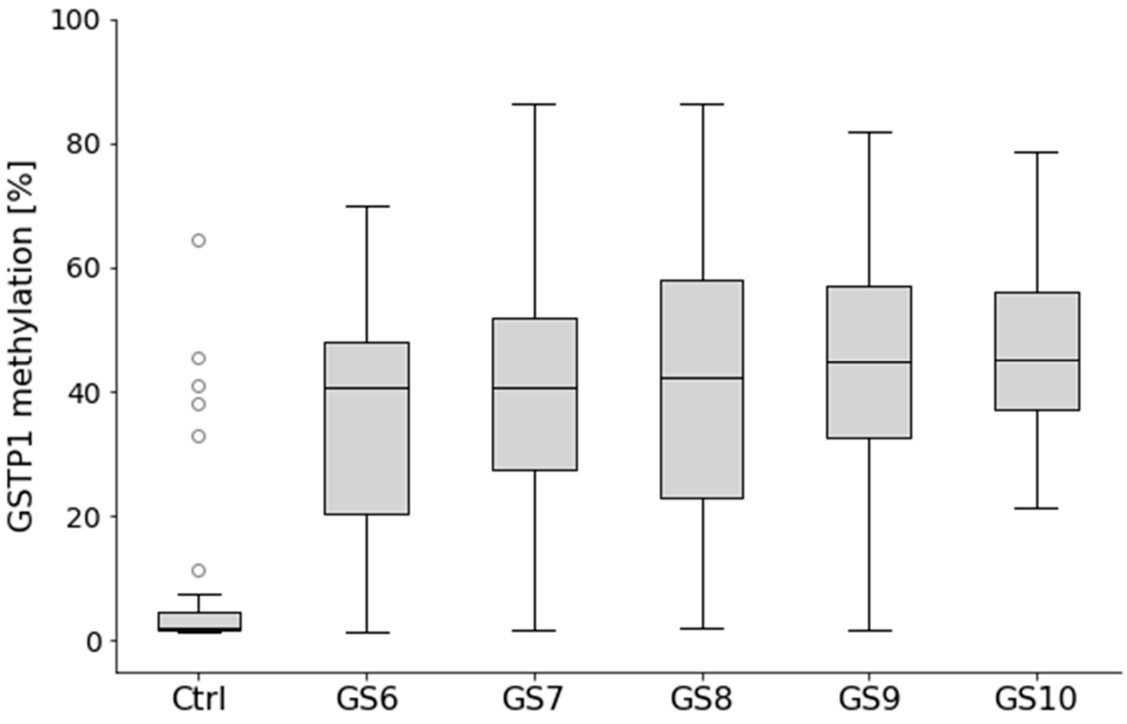

Supplementary Figure S1: *GSTP1* DNA methylation in prostate carcinoma biopsies compared to corresponding normal prostate tissue. Analyses are based upon data generated by the TCGA Research Network [34]. Prostate cancer patients were subdivided according to the Gleason score (GS) and compared to normal prostate tissue (Ctrl). GSTP1 methylation percentages represent the methylation status of the Infinium HumanMethylation450 BeadChip array probe cg02659086, which is located within the investigated OBBPA-ddPCR amplicon of this study.
